# Tumor immune-vascular crosstalk: synergy and translation of immune checkpoint inhibitors and anti-angiogenic agents in melanoma

**DOI:** 10.3389/fimmu.2026.1760044

**Published:** 2026-02-12

**Authors:** Yijie Xie, I. Ho, Zhipeng Liu, Keyu Chen, Minjie Zhou, Guodong Ha, Lincheng Duan, Zhengyu Zhao, Dingjun Cai

**Affiliations:** 1 Acupuncture and Tuina School, Chengdu University of Traditional Chinese Medicine, Chengdu, China; 2 Key Laboratory of Acupuncture for Senile Disease (Chengdu University of Traditional Chinese Medicine (TCM)), Ministry of Education/Acupuncture and Chronobiology Key Laboratory of Sichuan Province, Chengdu, China

**Keywords:** anti-angiogenic agents, combination therapy, immune checkpoint inhibitors, melanoma, tumor microenvironment

## Abstract

Melanoma is the most aggressive form of skin cancer. Although immune checkpoint inhibitors (ICIs) have led to major therapeutic breakthroughs, monotherapy remains limited by suboptimal response rates and pronounced resistance. In recent years, combination strategies integrating ICIs with anti-angiogenic agents have demonstrated substantial synergistic antitumor potential. This review systematically summarizes the mechanisms underlying this synergy, including cross-regulation between immune checkpoints and angiogenic factors (such as VEGF and ANG-2), the remodeling of the tumor immune microenvironment by anti-angiogenic agents, and feedback regulation of angiogenesis by ICIs. Preclinical studies indicate that such combinations can induce vascular normalization and enhance T-cell infiltration, thereby reversing immunosuppression. Subsequently, multiple clinical studies have confirmed that, compared with ICI monotherapy, combination therapy provides superior efficacy and acceptable safety in patients with advanced, mucosal, acral, and even brain-metastatic melanoma. Although the combined approach may increase adverse events such as cardiovascular complications and dermatologic toxicity, these risks can be controlled through multidisciplinary management. Overall, ICI-based combination therapy with anti-angiogenic agents represents a promising therapeutic paradigm for melanoma. Future research should focus on biomarker discovery and optimization of individualized precision strategies to maximize patient survival benefits.

## 1 Introduction

Melanoma, the most aggressive form of cutaneous malignancy, has shown a rapidly rising global incidence over the past five decades (1), and its onset and progression are closely associated with dysregulation of the immune system (2). In recent years, targeted therapies—most notably immune checkpoint inhibitors (ICIs) and anti-angiogenic agents—have provided new therapeutic options for patients with advanced melanoma (3). However, significant challenges persist in clinical practice: approximately 50% of patients exhibit no response to checkpoint inhibition (2), and disease progression remains common among those with metastatic melanoma even after receiving treatment (4) Patients with multifocal or disseminated lesions face particularly high mortality risks (4). These realities underscore the limitations of current therapeutic strategies and highlight the urgent need to develop novel combination treatment approaches.

Although ICIs (such as antibodies targeting PD-1, PD-L1, or CTLA-4) can reactivate immune pathways and promote melanoma rejection (5), their overall clinical efficacy remains suboptimal (6). Evidence shows that only a subset of patients benefits from ICI monotherapy (7), with substantial inter-individual variability in treatment response (8). Such variability is influenced by multiple factors—for example, mitochondrial dysfunction in tumor cells may affect their sensitivity to PD-1 inhibitors (6). Moreover, no reliable predictive biomarkers (e.g., tumor-associated antibodies) are currently available to accurately identify patients who are most likely to benefit from ICIs (9), further limiting the clinical application and efficacy improvement of monotherapy.

The combined use of anti-angiogenic agents and ICIs offers a new strategy to overcome existing therapeutic barriers in cancer treatment (10). The theoretical basis of this approach lies in their potential synergistic antitumor effects: ICIs block immune-escape signaling, while anti-angiogenic agents target the tumor-feeding vascular system (10). Studies have shown that anti-angiogenic agents can suppress the immunosuppressive features associated with angiogenesis and enhance antitumor immunity (7). This combination strategy is expected to increase the depth and durability of therapeutic responses, improve tumor control, and extend patient survival (11). This combination strategy is expected to increase the depth and durability of therapeutic responses, improve tumor control, and extend patient survival (3, 12).

Literature for this review was identified through searches of PubMed, Web of Science, and Embase up to November 2025. Key search terms included “melanoma”,”immune checkpoint inhibitors”, “anti-angiogenic therapy”, “VEGF”, “ANG2”, and “tumor microenvironment”. Both preclinical and clinical studies were considered, with emphasis on phase I–II trials, translational studies, and landmark mechanistic reports relevant to immune–vascular crosstalk. Non-English articles, single-agent monotherapy studies, case reports, reviews, or meta-analyses without original data were excluded. After title/abstract screening and full-text review, the most pertinent and high-quality evidence was synthesized to support the discussions in this review.

## 2 Synergistic mechanisms of combination therapy

### 2.1 Cross-regulation between ICIs and angiogenic factors

ICIs exert their effects by blocking signaling pathways that enable tumor cells to evade immune surveillance, whereas anti-angiogenic agents target the aberrantly activated tumor vasculature, restricting its supply of nutrients and oxygen (13). Extensive cross-regulatory interactions exist between the two at the molecular level. The vascular endothelial growth factor (VEGF) pathway has been identified as the “vascular counterpart” of immune checkpoints, and this structural analogy forms the theoretical basis for combination therapy (7). In melanoma, VEGF not only promotes angiogenesis but also directly suppresses T-cell function, whereas ICIs can reverse this immunosuppressive state (14). Members of the angiopoietin family, such as ANG-2, disrupt vascular stability, promote pathological angiogenesis, and act synergistically with VEGF to drive tumor progression (15). Furthermore, a bidirectional regulatory relationship exists between the PD-1/PD-L1 axis and VEGF signaling: PD-L1 expression can induce VEGF secretion, while VEGF can upregulate PD-L1 expression, forming a positive feedback loop that facilitates tumor progression (16, 17).

### 2.2 Immunomodulatory effects of anti-angiogenic agents on the tumor microenvironment

Anti-angiogenic agents can profoundly reshape the immunosuppressive characteristics of the tumor microenvironment. By inhibiting the VEGF or Ang2/Tie2 signaling pathways, these agents reduce the recruitment of regulatory T cells (Tregs) and myeloid-derived suppressor cells (MDSCs), while enhancing the infiltration and activation of cytotoxic T lymphocytes within tumor tissues (15, 18). Preclinical studies further demonstrate that anti-angiogenic therapy induces “normalization” of tumor vessel structure and function in melanoma models, improving intratumoral perfusion and oxygenation, thereby facilitating the trafficking and functional activity of immune effector cells (19). It is noteworthy that different classes of anti-angiogenic agents exert distinct regulatory effects on the immune microenvironment. For example, while lenvatinib and anti-VEGF antibodies both reduce intratumoral vascular density, their effects on the stability of endothelial cells within the blood–brain barrier differ (19).

### 2.3 Feedback regulation of angiogenesis by ICIs

**Figure 1:**
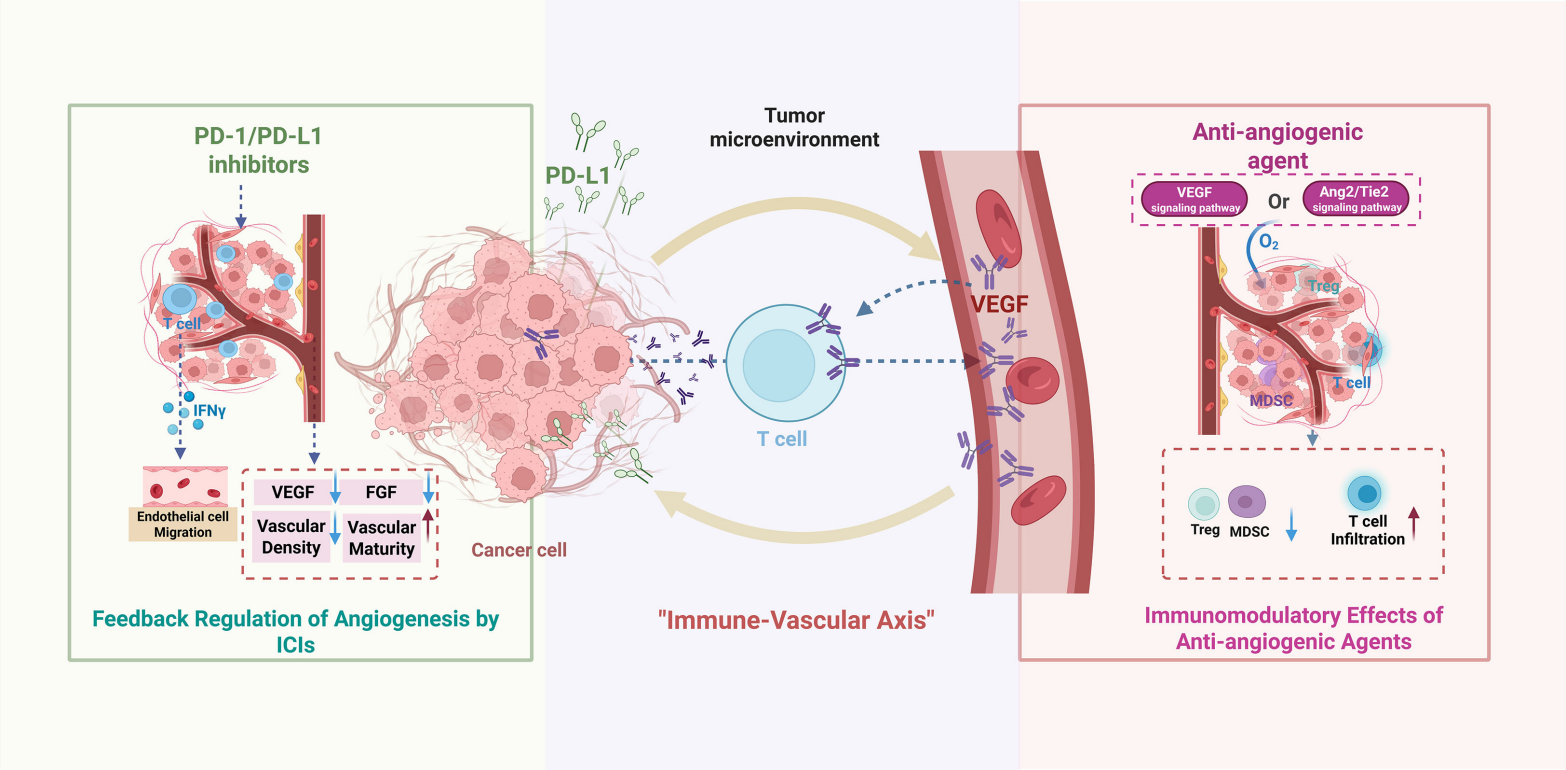
Synergistic mechanisms of combination therapy with icis and anti-angiogenic agents in tumor treatment. Created in biorender.

ICIs not only directly modulate the immune system but also regulate tumor angiogenesis through multiple mechanisms. PD-1/PD-L1 inhibitors can downregulate the secretion of pro-angiogenic factors—such as VEGF and FGF—by tumor cells and tumor-associated fibroblasts (16). In melanoma animal models, anti-PD-1 therapy decreases tumor vascular density and enhances vessel maturation, and this vascular normalization effect is closely associated with therapeutic response (19). Additionally, ICIs promote T-cell activation and stimulate the secretion of cytokines such as interferon-γ (IFN-γ), which further suppress endothelial cell proliferation and migration, thereby strengthening anti-angiogenic effects (20). Together, these mechanisms constitute a bidirectional “immune–vascular axis,” providing a strong scientific rationale for combination therapy (21). Recent studies also suggest that the gut microbiota may influence the efficacy of combination regimens by modulating angiogenesis and ICI-mediated antitumor activity, positioning it as a novel factor affecting treatment outcomes (3).

(**Figure 1** summarizes the mechanistic interplay between immune checkpoints and angiogenic pathways, the resulting microenvironmental remodeling, and the rationale for corresponding clinical combination strategies).

## 3 Evidence from preclinical studies

Multiple preclinical studies based on animal models have demonstrated clear synergistic antitumor effects of combining ICIs with anti-angiogenic agents in melanoma. In subcutaneous and left ventricular melanoma models, dual targeting of angiogenesis and immune checkpoints significantly improved control of both intracranial and extracranial disease (19). Further investigations revealed that in subcutaneous melanoma models, the VEGFR2-targeting anti-angiogenic agent DC101 optimized the tumor vascular microenvironment and immune-cell infiltration while upregulating PD-1/PD-L1 expression, thereby creating a tumor milieu favorable for subsequent combination treatment with ICIs (22). Another key mechanistic study focused on inhibitors of angiopoietin-2 (ANG-2). By targeting the ANG-2/TIE2 signaling axis, these inhibitors repair damaged vessels at the tumor margin, reduce vascular leakage, and promote vessel normalization, thereby removing the physical barriers that impede CD8^+^ T-cell infiltration into the tumor core. Simultaneously, treatment decreases Treg proportions and drives macrophage polarization toward the M1 phenotype, enhancing CD8^+^ T-cell cytotoxicity. Ultimately, ANG-2 inhibition synergizes with anti-PD-1 therapy to reverse immune resistance and improve therapeutic efficacy (23). In addition, the natural compound steppogenin (a 2’-hydroxyflavanone) has shown significant synergy when combined with anti-PD-1 antibodies. Steppogenin induces vascular normalization through modulation of the DLL4–NOTCH1 pathway, thereby improving T-cell infiltration and antitumor activity. This study not only validates the antitumor efficacy of the combination regimen but also provides preclinical evidence supporting the use of low-toxicity natural anti-angiogenic compounds in combination with ICIs (24). Collectively, these preclinical findings consistently indicate that anti-angiogenic therapy–induced vascular normalization can effectively improve the tumor immune microenvironment and enhance the response to ICIs, providing important theoretical support for the clinical translation of this combination strategy.

## 4 Clinical advances

### 4.1 Overview of published combination regimens

In recent years, combination regimens involving ICIs and anti-angiogenic agents have achieved substantial progress in the treatment of melanoma. Current clinical studies mainly focus on combining PD-1/PD-L1 inhibitors (such as nivolumab and pembrolizumab) or CTLA-4 inhibitors (such as ipilimumab) with VEGFR-targeting agents (such as bevacizumab and apatinib) or anti-Ang2 agents (such as MEDI3617) (25, 26). This strategy, which simultaneously blocks immune evasion pathways and tumor angiogenesis, has demonstrated synergistic efficacy superior to monotherapies (27).

The combination of the anti-PD-L1 inhibitor atezolizumab and the VEGFR monoclonal antibody bevacizumab achieved promising results in a phase II trial involving patients with advanced melanoma, with an objective response rate (ORR) of 45.0%, a disease control rate (DCR) of 65%, and a median progression-free survival (PFS) of 8.2 months (28). Safety was manageable, and subsequent three-year follow-up data further confirmed the durability of this regimen (29).

In a phase II trial evaluating bevacizumab plus pembrolizumab in treatment-naïve melanoma brain metastasis (MBM), 37 patients received four cycles of combined therapy every three weeks, followed by up to two years of pembrolizumab monotherapy. In this phase II non-randomized trial of treatment-naïve melanoma brain metastasis (MBM), the ORR reached 54.1%, median PFS was 1.2 years, and mOS was 4.3 years—results that require validation in large-scale phase III randomized controlled trials (RCTs). Higher baseline vascular density and smaller increases in circulating ANG-2 during treatment correlated with response. The combination was well tolerated and demonstrated significant efficacy in untreated MBM patients (30).

Numerous phase II studies have confirmed that ICIs combined with small-molecule TKIs targeting the VEGF/VEGFR pathway show remarkable potential in advanced melanoma. The phase II LEAP-004 study suggested that pembrolizumab combined with lenvatinib achieved an ORR of 21.4%, a PFS of 4.2 months, and an mOS of 14.0 months in patients with disease progression after prior ICI therapy, providing a potential second-line option for this refractory population. However, these results are preliminary and warrant confirmation in randomized controlled trials (31).

In resectable mucosal melanoma, which typically exhibits poor prognosis, neoadjuvant therapy with toripalimab (anti–PD-1) plus axitinib (anti-angiogenic agent) demonstrated notable efficacy: among 24 patients who underwent surgery, the ORR reached 33.3%, including 16.7% pathological complete responses and 16.7% pathological partial responses. This regimen significantly remodeled the tumor immune microenvironment, with marked increases in intratumoral CD3^+^ and CD3^+^CD8^+^ tumor-infiltrating lymphocytes, especially among patients who achieved pathological responses. The regimen was well tolerated, with grade 3–4 adverse events occurring in only 27.5% of patients and no treatment-related deaths, thus providing an effective and safe neoadjuvant option and laying groundwork for optimizing perioperative therapy for this melanoma subtype (32).

For advanced acral melanoma—an ICI-insensitive subtype with low tumor mutational burden(TMB)—the triplet regimen of camrelizumab (anti–PD-1), apatinib (VEGFR2 inhibitor), and temozolomide (chemotherapy) demonstrated substantial efficacy and acceptable safety as first-line therapy. The ORR reached 64.0%, and PFS reached 18.4 months, overcoming historical limitations in treatment effectiveness and emerging as a potential first-line therapeutic option for this ICI-insensitive subtype, supported by phase II data (33).

In a phase I trial, 46 patients with advanced melanoma received combined ipilimumab (anti–CTLA-4) and bevacizumab (anti-VEGF). The ORR was 19.6%, DCR was 67.4%, and mOS reached 25.1 months. Post-treatment tumor samples showed endothelial activation and increased infiltration of CD8^+^T cells and macrophages (34). Building upon this trial, another phase I study enrolled 15 patients treated with tremelimumab (CTLA-4 antibody) and the Ang2 inhibitor MEDI3617. Results demonstrated acceptable safety, with a DCR of 40% and an mOS of 15.4 months. Treatment was associated with increased circulating CD4^+^ and CD8^+^T cells expressing activation markers (ICOS^+^, HLA-DR^+^), along with elevated levels of immune-activating (IL-2) and immunoregulatory (IL-10) cytokines, suggesting robust antitumor immune activation and maintenance of immune homeostasis (26).

Collectively, these clinical findings indicate that most combination regimens exhibit significant efficacy with manageable toxicity, and some further enhance therapeutic benefit by remodeling the tumor immune microenvironment and activating antitumor immune homeostasis (**Table 1**).

**Table 1:** Clinical trials of ICIs and anti-angiogenic agents in melanoma.

Cancer type	Study design	Immune checkpoint inhibitor	Anti-angiogenic agents	Key results	Further analyses	Reference(s)
Mucosal Melanoma	Multicenter, Open-label, Single-arm, Phase II clinical trial	Atezolizumab	Bevacizumab	PFS: Preliminary Research:8.2 months 3-Year Follow-up:8.4 months OS: 3-Year Follow-up:23.7 months CI:29.3% – 61.5% ORR:45.0% DCR:65.0%		(28, 29)
Untreated Melanoma Brain Metastasis	Two-center, Open-label, Phase II clinical trial	Pembrolizumab	Bevacizumab	PFS:14.4 months mOS:51.6 months ORR:54.1%	smaller on-therapy increases in circulating ANG-2	(30)
Unresectable Stage III-IV Melanoma	Single-arm, Open-label Phase II clinical trial	Pembrolizumab	Lenvatinib	PFS:4.2 months mOS:14.0months ORR:21.4%		(31)
Resectable Mucosal Melanoma	Single-center, Open-label, Single-arm PhaseII clinical trial	Toripalimab	Axitinib	ORR:33.3%	a significant increase in CD3+ (P = 0.0032) and CD3+CD8+ (P = 0.0038) tumor-infiltrating lymphocytes after therapy	(32)
Treatment-naive unresectable stage III or IV acral melanoma	Single-center, Single-arm Phase II non-randomized clinical trial	Camrelizumab + Temozolomide (chemotherapy)	Apatinib	PFS:18.4 months ORR:64.0%		(33)
Measureable unresectable stage III or stage IV melanoma	Phase I clinical trial	Ipilimumab	Bevacizumab	mOS:25.1months ORR:19.6% DCR:65.0%	Increased endothelial activation and infiltration of CD8+ T cells and macrophages in tumor tissue after therapy	(34)
Metastatic or unresectable melanoma	Open-label, Phase I “3 + 3” dose escalation clinical trial	Tremelimumab	MEDI3617	mOS:15.4months DCR:40.0%	Increased numbers of activated CD4+ and CD8+ T cells (ICOS+, HLA-DR+) in circulation, accompanied by IL-2 and IL-10 production after therapy	(26)

### 4.2 Differences in the efficacy of various anti-angiogenic agents

Different VEGFR tyrosine kinase inhibitors (TKIs), such as apatinib, exhibit notable advantages in alleviating immune suppression within the tumor microenvironment by normalizing tumor vasculature and promoting T-cell infiltration (35). In contrast, anti-VEGF monoclonal antibodies (e.g., bevacizumab) primarily reduce tumor hypoxia and decrease the proportion of myeloid-derived suppressor cells (MDSCs) (36). In contrast, anti-VEGF monoclonal antibodies (e.g., bevacizumab) primarily reduce tumor hypoxia and decrease the proportion of myeloid-derived suppressor cells (MDSCs) (37). Anti-VEGF monoclonal antibodies have demonstrated significant overall survival (OS) benefits in non-small cell lung cancer (38), renal cell carcinoma (39), and have also shown encouraging activity in a phase II study of melanoma brain metastases (30). Meanwhile, anti-Ang2 agents (e.g., Ang2 inhibitors) can markedly enhance CD8^+^ T-cell infiltration into the tumor core, strengthening antitumor responses and reversing resistance to anti-PD-1 therapy (15, 23). This mechanism complements the T-cell-promoting effects of VEGFR-TKIs.

It is worth noting that Preclinical and translational studies suggest that treatment sequencing may influence efficacy; however, current evidence is largely indirect and derived from mechanistic models or non-melanoma settings, and optimal sequencing in melanoma remains to be prospectively defined (36).

In addition, the interpretation of these promising outcomes must also consider established clinical prognostic factors, such as serum lactate dehydrogenase (LDH) levels, which remain a cornerstone for risk stratification in advanced melanoma (40, 41). The promising yet variable clinical outcomes of ICI and anti-angiogenic agent combinations underscore the pressing need for predictive biomarkers to guide patient selection and sequence therapy. Here, we summarize key candidate biomarkers in **Table 2**.

**Table 2:** Candidate biomarkers for predicting response to combination therapy with ICIs and anti-angiogenic agents in melanoma.

Biomarker	Mechanism	Research hypothesis	Reference(s)
Circulating VEGF-A	High levels indicate angiogenic drive and immunosuppressive TME; baseline level may predict benefit from VEGF blockade.	High baseline circulating VEGF-A identifies a patient subset with strong “angiogenic immune suppression,” who would derive the greatest synergistic benefit from the addition of an anti-angiogenic agent to ICI therapy.	(14, 16, 17)
Circulating ANG-2	Dynamic changes on-treatment may reflect vascular normalization; smaller increase linked to better response in MBM (30).	The magnitude of early on-treatment changes in circulating ANG-2 serves as a dynamic, pharmacodynamic biomarker of vascular normalization and predicts long-term clinical outcome, particularly in melanoma brain metastases.	(15, 23, 30)
Endothelial activation signatures	Reflects tumor vascular dysfunction; correlates with T-cell infiltration barriers	Treatment-induced modulation of endothelial activation signatures in serial tumor biopsies correlates with increased CD8+ T-cell infiltration and can serve as an early histological biomarker of successful tumor microenvironment remodeling.	(19, 34)
T-cell–inflamed gene expression profile(GEP)/Tumor Mutational Burden(TMB)	T-cell–inflamed GEP reflects antitumor immune activity; TMB correlates with ICI responsiveness	High T-cell–inflamed GEP + moderate TMB enhances synergy (anti-angiogenics reduce immune exclusion)	(15, 18, 33)
Lactate Dehydrogenase(LDH)	Elevated LDH is a poor prognostic factor in advanced melanoma; may identify patients with high tumor burden/metabolic dysregulation.	The efficacy of ICI/anti-angiogenic combination therapy is less compromised by elevated baseline LDH compared to ICI monotherapy, due to the combination’s direct targeting of hypoxia and vascular dysfunction.	(40, 41)

## 5 Safety analysis and management of adverse events

### 5.1 Toxicity profile of combination therapy

Combination therapy with ICIs and anti-angiogenic agents, while providing synergistic antitumor effects, is also associated with unique toxicity characteristics. The increased toxicity largely arises from overlapping mechanisms: ICIs can induce autoimmune toxicities by releasing immune inhibition (42), whereas anti-angiogenic agents may lead to vascular-related adverse effects (43). Clinical studies (44) indicate that adverse events (AEs) occur more frequently with combination therapy than with PD-1 monotherapy, but the spectrum of toxicities (hepatic, endocrine, dermatologic, etc.) is similar to those observed with either agent alone, without introducing new or unexpected toxicities.

Cardiovascular toxicity is a particularly significant concern. Real-world evidence suggests that combining the two classes of drugs increases the incidence of hypertension, myocarditis, and heart failure (45). A meta-analysis of randomized controlled trials found that anti-VEGF drugs (e.g., bevacizumab) are associated with increased risk of multiple cardiovascular events, while ICIs alone do not markedly raise cardiovascular risk (46). ICI-related cardiovascular toxicities mainly include acute myocarditis, chronic inflammatory cardiomyopathy, and ischemic heart disease (47, 48), whereas anti-angiogenic agents (e.g., TKIs) disrupt vascular homeostasis, leading to hypertension, thrombosis, and elevated bleeding risk (49). Combination therapy significantly increases the incidence of hypertension, thrombotic events, and bleeding, further worsening these toxicities (45, 50).

Dermatologic toxicity is another common category of adverse events (51). Skin toxicities with ICI monotherapy—such as rash, pruritus, and depigmentation—occur in about 20–30% of patients (52, 53), lower than the 65.3% observed with combination therapy. This suggests that anti-angiogenic agents may exacerbate mucocutaneous irritation, leading to a higher incidence of skin toxicity (44).

### 5.2 Monitoring and management of special adverse events

For adverse events uniquely associated with combination therapy, targeted monitoring and management strategies are required. In the cardiovascular system, enhanced surveillance of cardiac enzymes, electrocardiograms, and cardiac imaging is recommended during treatment, with prompt intervention upon detection of abnormalities (45, 54). Hypertension, a common AE, should be managed with angiotensin-converting enzyme inhibitors or calcium channel blockers (45). A hepatocellular carcinoma study suggests evaluating baseline cardiovascular risk when combining ICIs with anti-angiogenic agents (55).

Management of dermatologic toxicities requires differentiating between types of skin involvement. For immune-related skin toxicity, mild rashes may be treated with topical corticosteroids, whereas moderate-to-severe rashes may require pausing immunotherapy and implementing systemic corticosteroids (53, 56). Importantly, cutaneous manifestations such as vitiligo may correlate with favorable treatment response and should not automatically prompt discontinuation of effective therapy (56). Studies show that patients who develop cutaneous irAEs respond better to ICIs (57), though the severity and prognostic implications of skin toxicities in combination therapy warrant further investigation. Early recognition and intervention (e.g., topical or systemic immunosuppression) are crucial for preserving quality of life.

For predicting and mitigating immune-related inflammation, patients with pre-existing autoimmune diseases (pAID) have significantly higher rates of irAEs and require enhanced autoantibody monitoring (58). Moreover, baseline peripheral inflammatory cytokine profiles—such as elevated IL-23—may predict risk of severe irAEs (59).

Regarding optimization of anti-angiogenic drug dosing and the “vascular normalization window,” precise dose adjustments are needed: insufficient dosing may promote angiogenesis, whereas excessive dosing inhibits immune-cell infiltration (7, 60). Imaging-based monitoring of tumor vascular structure (e.g., perfusion parameters) may help identify the normalization window, during which administering ICIs can reduce irAE risk (7, 60). Furthermore, bleeding risk associated with anti-angiogenic agents requires particular attention—especially in patients with brain metastases (61). These patients also need monitoring for neuroinflammation (e.g., microglial activation markers), which may relate to ICI efficacy and neurotoxicity (62, 63). Lower starting doses and extended monitoring intervals are advised for elderly patients or those with autoimmune histories (64).

Throughout treatment, establishing a multidisciplinary team—including oncology, cardiology, and dermatology specialists—is recommended to manage complex adverse events (64). Patient education should also be strengthened to ensure early recognition and timely reporting of symptoms. These integrated measures help maximize the clinical benefits of combination therapy while ensuring patient safety.

## 6 Conclusion

Combination regimens involving immune checkpoint inhibitors (ICIs) and anti-angiogenic agents represent a highly promising new paradigm in the treatment of melanoma. This review highlights that the core advantage of this strategy lies in its ability to remodel the tumor immune microenvironment through the synergistic modulation of the “immune–vascular axis,” thereby promoting vascular normalization and enhancing T-cell infiltration. These effects help overcome therapeutic resistance and improve treatment efficacy. Clinical studies have demonstrated that such combination strategies achieve superior efficacy and safety compared with monotherapies across multiple challenging melanoma subtypes, including advanced, mucosal, acral melanoma, and brain metastases.

Despite its promising outlook, several key challenges remain in this field:

1. Most current clinical evidence is derived from phase II studies and small-sample trials, lacking validation from large-scale phase III randomized controlled trials. Therefore, the long-term efficacy and safety of this strategy require further confirmation.
2. Optimal sequencing and dosing regimens for different drug combinations remain undefined, necessitating further optimization.
3. Combination therapy may increase the risk of adverse events, particularly cardiovascular and dermatologic toxicities, imposing higher demands on clinical monitoring and management.

Future research should aim to advance the precision and individualization of this therapeutic strategy. Key directions include: conducting in-depth mechanistic studies to identify biomarkers that can predict efficacy and toxicity (such as specific angiogenic factors, immune cell subsets, or microbiome signatures) and integrating them with established clinical prognostic factors (e.g., serum lactate dehydrogenase, LDH); designing rigorous, large-scale clinical trials to establish optimal therapeutic models; and implementing multidisciplinary collaborative management systems in clinical practice. Special attention should be given to monitoring and dose adjustment for vulnerable patient populations. Through these approaches, it will be possible to control toxicity while maximizing survival benefits, ultimately leading to the maturation and standardization of this highly promising therapeutic strategy.
